# Effect of different cryopreservation media on human nucleus pulposus cells' viability and trilineage potential

**DOI:** 10.1002/jsp2.1140

**Published:** 2021-02-14

**Authors:** Andreas S. Croft, Julien Guerrero, Katharina A. C. Oswald, Sonja Häckel, Christoph E. Albers, Benjamin Gantenbein

**Affiliations:** ^1^ Tissue Engineering for Orthopaedics and Mechanobiology (TOM), The Department for BioMedical Research (DBMR) of the Medical Faculty of the University of Bern University of Bern Bern Switzerland; ^2^ Department of Orthopaedic Surgery and Traumatology, Inselspital, Bern University Hospital University of Bern Bern Switzerland

**Keywords:** biological therapies, cryopreservation, culture media, stem cell, trilineage differentiation

## Abstract

**Introduction:** Low back pain (LBP) is a significant cause of disability in many countries, affecting more than half a billion people worldwide. In the past, progenitor cells have been found within the nucleus pulposus (NP) of the human intervertebral disc (IVD). However, in the context of cell therapy, little is known about the effect of cryopreservation and expansion on here called “heterogenic” human NP cells (hNPCs), and whether commercially available cryopreservation media are more efficient than “commonly used” media in terms of cell viability.

**Materials:** In this study, hNPCs from four trauma patients (age 40.5 ± 14.3 years) and two patients with degenerated IVDs (age 24 and 46 years), undergoing spinal surgery, were collected. To isolate hNPCs, the tissue was digested with a mild two‐step protocol. After subsequent expansion, hNPCs at passages 2‐5 were separated and either cryo‐preserved for 1 week at −150°C or differentiated into osteogenic, adipogenic, or chondrogenic lineages for 21 days. Cryopreservation was performed with five different media to compare their effect on the cell's viability and differentiation potential. Cell viability was determined with flow cytometry using propidium iodide and the trilineage differentiation potential was assessed by quantitative polymerase chain reaction and histological analysis.

**Results:** After 1 week of cryopreservation, the hNPC's cell viability was comparable for all conditions, that is, independent of the cryopreservation medium used (82.3 ± 0.8% of cell viability). Furthermore, hNPCs from trauma patients showed some evidence for adipogenic and chondrogenic differentiation and at lower levels, this and evidence of osteogenic differentiation could be confirmed with hNPCs from degenerated discs. Moreover, cryopreservation did not affect the cell's differentiation potential in the majority of the cases tested.

**Conclusion:** “Commonly used” cryopreservation media seem to perform just as well as commercially available media in terms of cell viability and the overall maintenance of the hNPCs trilineage differentiation potential.

## Graphical abstract

Human nucleus pulposus cell populations (hNPCs) were isolated, expanded, and then cryo‐preserved at −150°C with one of five different cryopreservation media and/or the cells' trilineage differentiation potential was assessed. The cells' viability did not differ between the cryopreservation media tested and hNPCs showed evidence for osteogenic, adipogenic and chondrogenic differentiation.

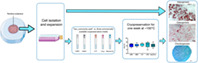

## 1 INTRODUCTION

### 1.1 Intervertebral disc degeneration and low back pain

A healthy intervertebral disc (IVD) mainly consists of a centrally located, collagen type II rich and highly hydrated nucleus pulposus tissue (NP). The NP is surrounded by the annulus fibrosus (AF), which is composed of 15 to 25 concentric lamellae consisting of collagen type I and II fibers with elastin fibers lying in between. Finally, the IVD is enclosed by two hyaline cartilaginous endplates (CEP) above and below them.^1^, ^2^, ^3^

The causes of IVD degeneration (IDD) are manifold. However, in many cases, IDD is initiated by a decrease of the amount of NP cells accompanied by a change of the extracellular matrix (ECM) composition, for example, by loss of the proteoglycan content.^4^ Degraded proteoglycans tend to escape from the NP and osmotic imbalance results as a consequence.^5^ This reduction of the osmotic pressure in the NP will then lead to its dehydration and destruction, resulting in loss of disc height.^6^ Other causes of IDD are changes in collagen repartitions in the ECM. Especially type II collagen can become more and more denatured over time and therefore contribute to IDD.^7^, ^8^

While IDD per se may remain clinically silent, some conditions associated with IDD cause symptoms ranging from mild, occasional discomfort to severe, immobilizing back pain and disability.^9^ Conservative management, including physical therapy, lifestyle modification, pain‐ and anti‐inflammatory medication is the first‐line treatment of back pain related to IDD. Failure of conservative measures may direct towards surgical treatment.^10^, ^11^ The spectrum of surgical procedures is broad and relies on distinct pathologies. One of the most frequently performed surgical intervention is spinal fusion with the purpose to induce osseous fusion between at least two vertebrae and thereby eliminating motion and relieving pain.^12^ However, the clinical success rate of spinal fusion lies only between 50% and 70%, and more than 25% of the patients require reoperation, which still does not guarantee successful fusion.^13^, ^14^, ^15^ Another primary concern of spinal fusion is that these interventions do not have the intention to restore the IVD, but instead leave the patient with a stiff and immobile spine, increasing the risk for adjacent segment disease. Therefore, there is a high demand for new efficient treatment techniques regarding LBP.

### 1.2 Cell‐based therapy for IDD

A promising approach to regenerate the IVD is cell‐based therapy. Even if it is still in its infancy, cell‐based therapy has received more and more attention over the last two decades.^2^ Especially targeting the NP is believed to have considerable potential in this field.^2^ A previous study showed the existence of NP derived mesenchymal stromal cells (MSCs) that can differentiate into an osteogenic and chondrogenic lineage. However, they were not able to differentiate into adipocytes.^16^ Additionally, over the last decade, progenitor cells positive for Tie2 (aka angiopoietin‐1 receptor / TEK receptor tyrosine kinase) and disialoganglioside 2 (GD2^+^) have been found in the NP of humans and other vertebrates.^17^, ^18^ It has been shown that these rare cells can form spheroid colonies in methylcellulose‐based medium, and they are also able to undergo trilineage differentiation.^17^ However, with increasing age and continuous IDD, the amount of Tie2 positive cells in the IVD rapidly decreases. In humans, the frequency of these cells already starts to drop before the age of 20, with more than half of the NP cells usually being positive for Tie2.^17^ By the age of 50, the amount of Tie2 positive cells nearly completely vanishes.^17^, ^19^

To conclude, the differentiation potential of MSCs and Tie2 positive cells from the NP has been demonstrated in recent years. However, little is known about the effect of expansion and cryopreservation on here called “heterogenic” human NP cell populations (hNPCs) and their stemness in the context of cell therapy for regeneration of the IVD.^19^ Thus, we hypothesized that “heterogenic” hNPCs could undergo trilineage differentiation. Therefore, freshly isolated hNPCs and previously frozen hNPCs (from the same donors) were cultured either in osteogenic inductive medium, adipogenic inductive medium, chondrogenic inductive medium, or control medium. After 21 days of culture, multi‐lineage differentiation properties were compared using histology, absorbance measurements, and quantitative polymerase chain reaction (qPCR).

### 1.3 Cryopreservation

Cryopreservation has become an indispensable technology for biomedical research but also clinical medicine and it is even seen as the gold standard for bio‐preservation.^20^, ^21^ However, freezing cells at very low temperatures (−150°C) for preservation faces many challenges in terms of cell viability. For example, the freezing rate plays a critical role in successful cryopreservation as it underlies a delicate balance between too fast and too slow cooling.^20^ If the cooling process happens too fast, liquid water in the cell's body will undergo a phase change and form ice crystals which potentially damage the cells.^22^ In contrast, a too slow cooling rate will first cause extracellular ice formation and as a consequence create an osmotic imbalance between the intracellular and extracellular space. As more and more extracellular ice forms, the concentration of particles in the residual water will further increase causing greater osmotic imbalance and an additional depression of the freezing point.^23^, ^24^ As a result, intracellular water will efflux across the cell's membrane leading to dehydration and in the worst case cause cell death.^25^

To avoid this ice crystal formation and to preserve osmotic balance, a cryoprotectant is added to the Mammalian cells and then everything is usually cooled at a rate of −1°C per minute.^26^ By definition, a cryoprotectant is a solute added to the medium that enables higher cell recovery after thawing compared to its absence.^27^ Many different cryoprotectants exist, one of which being dimethyl sulfoxide (DMSO), which is most commonly used for mammalian cells.^28^, ^29^ DMSO can cross the cell membrane and act as a solvent for salts. As a consequence, the osmotic pressure diminishes, the freezing point decreases, and intracellular ice crystal formation is depressed.^24^, ^26^

In the past, studies have shown that cryopreservation of human adipose‐derived mesenchymal stromal cells (ASCs) and bone marrow‐derived mesenchymal stromal cells (BMSCs) does not affect their viability nor their trilineage differentiation potential.^30^, ^31^ However, an optimal way to cryo‐preserve hNPCs is yet to be found for IVD cells in general and only very little is known about the influence of cryopreservation on the cell viability and stemness of hNPCs.

Thus, we hypothesized that hNPCs can be cryo‐preserved just as well with “commonly used” cryopreservation media, made by simply adding 10% dimethyl sulfoxide (DMSO), as with more complex commercially available media without any significant differences concerning cell viability. Additionally, we wondered if hNPCs can undergo trilineage differentiation and if cryopreservation would affect this ability. Therefore, primary hNPCs were isolated from either trauma patients or patients suffering from degenerated IVDs undergoing spinal surgery. We cryopreserved the isolated hNPCs for 2 days at −80°C followed by 1 week's storage at −150°C with five different cryopreservation media. After testing the cell viability using flow cytometry, the hNPCs' trilineage differentiation potential was assessed.

## 2 MATERIALS AND METHODS

### 2.1 Cell isolation

NP cells were isolated from human IVDs, which were obtained from donors undergoing spinal surgery because of IDD (N = 2) or after trauma, whereby IVDs should not be degenerated or at most only mildly (N = 4) (Table 1). All patients provided written informed consent after the project was approved by the local IRB (ethics committee of the Canton of Bern, Ref. 2019‐00097). The IVD tissue was then dissected into nucleus pulposus (NP) tissue, annulus fibrosus (AF) tissue, and cartilaginous endplate (CEP) tissue. In the next step, the prepared pieces of each IVD tissue type were washed with phosphate‐buffered salt solution (PBS) and centrifuged for 5 minutes at 500*g*. Afterwards, all three tissue types were first digested with 0.2% pronase (#10165921001; Roche Diagnostics, Mannheim, Germany) for 1 hour and subsequently digested with collagenase type II (255 U/mg; #LS004176; Worthington Biochemical Corporation, Lakewood, New Jersey) overnight. After overnight digestion, the cells were washed with PBS, transferred into T150 flasks (#90151; TPP, Trasadingen, Switzerland) and expanded in LG‐DMEM “complete medium” (low glucose Dulbecco's Modified Eagle Medium (1 g/L; LG‐DMEM; #31600‐083; Thermo Fisher Scientific, Waltham, Massachusetts) enriched with 0.22% sodium hydrogen carbonate (#31437‐500G‐R; Sigma‐Aldrich), 10% FBS (#F7524; Sigma‐Aldrich), 1 mM sodium pyruvate (#11360‐039; Thermo Fisher Scientific), penicillin/streptomycin/glutamine (#10378‐016; P/S/G, 100 units/mL, 100 μg/mL and 292 μg/mL, respectively; Thermo Fisher Scientific) and 10 mM HEPES buffer solution (#15630‐056; Thermo Fisher Scientific). Cells from the AF the CEP were frozen and the “heterogenous” hNPC population was expanded until a sufficient number of cells were accomplished for future experiments. During cell expansion, the medium was changed twice a week. Cells were cultured at 37°C with normoxic conditions (20% O_2_) and 5% CO_2_.

**TABLE 1:** Overview of the sex, age, disc location, status of the received IVD and Pfirrmann grade (if degenerated) from all patients in this study

Donors of the study				
Donor number	Sex	Age [years]	Disc location	IVD status (Pfirrmann)
#1	Male	50	Th11/Th12 & L1/L2	Trauma
#2	Male	55	Th11/Th12 & L1/L2	Trauma
#3	Male	32	L1/L2	Trauma
#4	Female	24	L5/S1	Degenerated (Grade 4)
#5	Female	46	L5/S1	Degenerated (Grade 4)
#6	Female	25	Th12/L1 & L1/L2	Trauma

Abbreviations: IVD, intervertebral disc; L, lumbar; S, sacral; Th, thoracic.

### 2.2 Cell freezing

To find the most suitable cryopreservation medium for hNPCs, two “commonly used” and three commercially available cryopreservation media were tested. For the “commonly used” media we used either a commonly applied cryo‐medium for progenitor cells (CMPC) that consisted of 90% FBS and 10% DMSO or a commonly applied cryo‐medium for differentiated cells (CMDC) that consisted of 90% LG‐DMEM “complete medium” and 10% DMSO. In addition, three commercially available cryopreservation media were tested. One of them was the CellnTec Cryo‐Defined Freezing Medium (CnT) (#CnT‐CRYO‐50; CELLnTEC Advanced Cell Systems Inc.; Bern, Switzerland), which was mixed with 50% LG‐DMEM “complete medium” according to the product usage requirements. The second commercially available cryopreservation medium we tested was the cGMP‐manufactured CryoStor CS10 (CS10) (#07930; Biolife Solutions; Bothell, Washington) and as a last tested medium, we took the MesenCult‐ACF Freezing Medium (MesenCult) (#05490; Stemcell Technologies; Vancouver, British Columbia, Canada).

The hNPCs were frozen at a density of 500 000 cells/mL of the respective cryopreservation medium. In brief, the cell suspensions were first stored in a pre‐cooled Nalgene Cryo 1°C freezing container (#5100‐0001; Thermo Fisher Scientific) filled with 2‐propanol for 2 days at −80°C to reduce ice crystal formation. Then, the cells were removed from the freezing container and stored for 7 days at −150°C.

### 2.3 Cell thawing

To maximize hNPCs' viability, subsequent procedures were followed: First, LG‐DMEM “complete medium” was put into a prewarmed water bath (37°C). As soon as the medium reached the same temperature as the water bath, the samples were removed from the −150°C freezer. Under sterile conditions, the cryovial's caps were twisted a quarter‐turn to relieve internal pressure and then retightened. Then they were moved into the prewarmed water bath and gently shaken until only a small ice pellet remained in the vials. Afterwards, the cryovials were sprayed with ethanol (80%), and under sterile conditions, the cell suspensions were then diluted 1:10 with warmed LG‐DMEM “complete medium” followed by centrifugation (500 g for 5 minutes). After centrifugation, the supernatant was discarded and the cells were resuspended with LG‐DMEM “complete medium”. To reach a sufficient number of cells for trilineage differentiation, the cells were first expanded in LG‐DMEM “complete medium” after the thawing process.

### 2.4 Cell viability

To assess the most effective cryopreservation medium for NP cells in terms of cell viability, hNPCs' viability was assessed by automatic counting using flow cytometry for dead cells stained with propidium iodide (#P4864‐10ML; Sigma‐Aldrich). Therefore, thawed hNPCs were put into suspension with a buffer for flow cytometry (PBS, 0.5% Human Serum Albumin, 0.5 mM EDTA [Sigma‐Aldrich]) and then stained with propidium iodide (#P4864‐10ML; Sigma‐Aldrich). After incubation (30 minutes at 4°C), the cell suspensions were centrifuged (500 g for 5 minutes) and washed twice with the buffer for flow cytometry. For each condition, a minimum of 10 000 events was recorded with the Cytoflex (Beckman Coulter; Brea) that was used for flow cytometry. The following gating strategies were applied for flow cytometry: All cells were first gated for whole‐cell populations. Therefore, the forward scattered height (FSC‐H) was plotted against the side scattered height (SSC‐H). In a next step, to discriminate doubles from single cells, the side scattered area (SSC‐A) was plotted against the SSC‐H.

### 2.5 Trilineage differentiation

#### 2.5.1 Overview

To test the multipotent potential of hNPCs, freshly isolated hNPCs at passages 2 to 3 were driven to undergo osteogenesis, adipogenesis, and chondrogenesis after subsequent expansion. Additionally, to assess the influence of cryopreservation and the effect of the cryopreservation medium itself on the multipotent potential of hNPCs (passages 3‐5) that were previously frozen in one of five different cryo‐protectants were also driven to undergo trilineage differentiation. Trauma and degenerated samples were analyzed separately due to their different composition and general state. Degenerated tissue is known to have an altered composition of the ECM, is osmotically imbalanced, and contains increased levels of proinflammatory cytokines.^4^, ^5^, ^32^

#### 2.5.2 Osteogenic differentiation

To test the osteogenic differentiation potential of hNPCs, cells were seeded on a 12‐well plate (#92012; TPP) with a density between 10 000 and 15 000 cells/cm^2^ and cultured either with control or osteogenic inductive medium for 21 days. The control medium consisted of α ‐ Modified Eagles Medium (α ‐ MEM; #12000‐063; Thermo Fisher Scientific) containing 10% FBS, 1 mM sodium pyruvate, penicillin/streptomycin/glutamine (P/S/G, 100 units/mL, 100 μg/mL and 292 μg/mL, respectively) and 10 mM HEPES buffer solution (aka. α ‐ MEM „complete medium“). The osteogenic inductive medium consisted of the same ingredients as the control medium with the addition of 100 nM dexamethasone (#D1756), 10 mM 𝛽‐glycerophosphate (#G9422), and 100 μM L‐ascorbic acid 2‐phosphate (#A8960; all from Sigma‐Aldrich). Medium change was performed twice a week.

#### 2.5.3 Adipogenic differentiation

To investigate the adipogenic differentiation potential of hNPCs, they were seeded on a 12‐well plate (#92012; TPP) with a density between 10 000 and 15 000 cells/cm^2^ and cultured either with a control medium (α ‐ MEM „complete medium“) or an adipogenic inductive medium for 21 days. The adipogenic inductive medium consisted of the same components as the control medium with the addition of insulin (10 μg/mL; #I0516), 1 μM dexamethasone (#D1756), isobutylmethylxanthine (IBMX; 10 μg/mL; #I7018), and 100 μM indomethacin (#I7378; everything from Sigma‐Aldrich). Also, medium change was performed twice a week.

#### 2.5.4 Chondrogenic differentiation

The chondrogenic differentiation potential was tested with hNPCs cultured as 3D pellets. Therefore, between 250 000 and 300 000 hNPCs were centrifuged for 5 minutes at 500 g to a cell pellet and cultured either with a control medium (α ‐ MEM „complete medium“) or chondrogenic inductive medium. The chondrogenic inductive medium comprised high glucose Dulbecco's Modified Eagle Medium (3.7 g/L; HG‐ DMEM; #52100‐039; Thermo Fisher Scientific) supplemented with 1 mM sodium pyruvate, penicillin/streptomycin/glutamine (P/S/G, 100 units/mL, 100 μg/ mL and 292 μg/mL, respectively), 10 mM HEPES buffer solution, 100 nM dexamethasone (#D1756; Sigma‐ Aldrich), 1% insulin + transferrin + selenium (ITS), 1% bovine serum albumin (BSA) and linoleic acid (#I2521‐5ML), 0.1 mM L‐ascorbic acid‐2‐phosphate (#A8960; all from Sigma‐Aldrich), 1% minimum essential medium non‐essential amino acid (MEM NEAA; #11140‐035; Thermo Fisher Scientific) and 10 ng/mL TGF𝛽‐3 (#243‐B3‐002/CF; R&D Systems; Minneapolis). The pellets were cultured for 21 days and the medium was changed twice a week.

### 2.6 Histology

Histological staining was performed for the trilineage differentiation assays after 21 days of culture. Following steps were performed:

#### 2.6.1 Osteogenic lineage

First, hNPCs were washed three times with PBS. Then, the cells were fixed with 4% formalin (#252549‐1 L, Sigma‐Aldrich) and then incubated overnight at 4°C. Afterwards, cells were rinsed twice with distilled water and 2% Alizarin red solution was added. After an incubation time of 45 minutes in the dark at room temperature, the cells were rinsed four times with distilled water and finally covered with PBS. The Alizarin red staining consisted of 2% Alizarin red powder (#A5533‐25G; Sigma‐Aldrich) dissolved into distilled water.

#### 2.6.2 Adipogenic lineage

Here, cells were washed twice with PBS. Afterwards, they were fixed with 60% 2‐propanol for 2 minutes at room temperature and replaced with Oil‐Red‐O working solution. After 20 minutes of incubation time, the cells were washed twice with PBS and finally covered with PBS. The Oil‐Red‐O working solution was made of three parts Oil‐Red‐O stock solution and two parts distilled water. The Oil‐Red‐O stock solution consisted of 0.375% Oil‐Red‐O powder (#1.05230; Merck; Darmstadt, Germany) dissolved into 2‐propanol (#278475‐1 L; Sigma‐Aldrich).

#### 2.6.3 Chondrogenic lineage

At the end of the differentiation assay, the 3D pellets were washed with PBS, fixed overnight in a 4% formalin solution at 4°C. The next day, the formalin was replaced with PBS and the pellets of each condition were embedded into HistoGel (#HG‐4000‐012, Thermo Fisher Scientific) and transferred into histological cassettes (#81‐0103‐00; Sanowa; Leimen, Germany), where they were dehydrated and processed overnight with the STP 120 Spin Tissue Processor (#813150; Thermo Fisher Scientific). After overnight dehydration, the samples were embedded into paraffin and then cut into histological sections (7 μm thickness). In a next step, the samples were either stained with Alcian blue (1% Alcian blue 8GX; #27221) or Safranin‐O (0.1% Safranin‐O; #1.15948; Merck) including a counter‐stain with Fast green powder (0.02% Fast Green FCF; #1.04022; Merck) diluted in distilled water. Finally, all samples were mounted using a coverslip and with Eukitt (#03989‐100ML; O. Kindler Inc.; Freiburg, Germany).

### 2.7 Quantification of the trilineage differentiation assay

#### 2.7.1 Quantification of the Alizarin red staining content

To quantify the amount of Alizarin red staining present in the 12‐well plates, we put the stained matrix back into solution. Therefore, the H_2_O was removed from all wells and replaced with 10% cetylpyridinium chloride in 10 mM NaPO4 (#71645‐1KG; Fluka). The 12‐well plates were then put on an orbital shaker for 1 hour until all the stain was solubilized. Afterwards, all samples were pipetted into a 96‐well plate, and then the absorbance was read with a wavelength of 570 nm (SpectraMax M5; Bucher Biotec, Basel, Switzerland).

#### 2.7.2 Quantification of the Oil‐Red‐O staining content

After staining, the Oil‐Red‐O positive signal in the 12‐well plates had to be put into solution for quantification. Therefore, the PBS was removed and all wells were air‐dried. Afterwards, 100% 2‐propanol was added to each well and put on an orbital shaker for 15 minutes. The extracted Oil‐Red‐O staining was then transferred into the wells of a 96‐well plate and absorbance was measured with a wavelength of 500 nm (SpectraMax M5).

#### 2.7.3 Quantification of the glycosaminoglycan content in the Supernatant

To study the chondrogenic differentiation of the 3D pellets, the concentration of glycosaminoglycans (GAG) in the supernatant was measured at day 21 of cell culture, with the last exchange of medium being carried out 4 days before the end of the culture. Therefore, a stock solution of dimethylmethylene blue (DMMB) was prepared, consisting of 0.0021% 1.9‐dimethyl‐methylene blue zinc chloride double salt (#341088, Sigma‐ Aldrich), 0.2% sodium formate (#71541, Fluka), absolute ethanol, and distilled water. By adding formic acid, the pH was adjusted to 1.5. The DMMB was then added to the supernatant of the respective samples and within 5 minutes, the absorbance could be recorded at a wavelength of 595 nm (SpectraMax M5).

### 2.8 Quantitative polymerase chain reaction

Total RNA was extracted using the GenElute Mammalian Total RNA Miniprep Kit (#RTN70‐1KT; Sigma‐ Aldrich). In contrast to the cells from the adipogenic and osteogenic differentiation assays, the 3D pellets from the chondrogenic differentiation assays first had to be lysed with a tissue lyser (Retsch Inc.; Haan, Germany) before extracting the RNA. Reverse transcription into cDNA was performed with the 5x qRT SuperMix (#B24404, Bimake, San Francisco, California) together with the MyCycler thermal cycler (#170‐9703, Bio‐Rad Laboratories; Hercules, California). The cycling parameters were: 25°C for 5 minutes, followed by 42°C for 30 minutes, and then 85°C for 5 minutes. Real‐time quantitative polymerase chain reaction (qPCR) was performed with the CFX96 Real‐Time System (#185‐5096; Bio‐Rad Laboratories) using the primers of interest (Table 2) together with the 2x SYBR green master mix (#B21202; Bimake) undergoing a two‐step protocol for 45 cycles with a denaturation temperature of 95°C for 15 seconds and a combined annealing and extension at 61°C for 30 seconds. To determine the gene expression, the threshold cycles (C_t_) of the different genes were analyzed and the ∆∆Ct was calculated using GAPDH as a reference gene. In the end, 2^‐∆∆Ct^ was calculated to get the fold change of each gene. Samples with a Ct above 35 cycles or with an unconventional melting curve were excluded.

**TABLE 2:** Overview of all genes used for qPCR using Glycerinaldehyde 3‐phosphate dehydrogenase (GAPDH) as a reference gene. Genes analyzed for the chondrogenic lineage: SOX‐9, aggrecan (ACAN), collagen type 2 (COL2), collagen type 1 (COL1) and collagen type 10 α 1 (COL10). For the osteogenic lineage, following genes were analyzed: Collagen type 1 (COL1), runt‐related transcription factor 2 (RUNX2), osteocalcin (OCN), osteopontin (OPN), osterix (OSX) and alkaline phosphatase (ALP). Genes analyzed for the adipogenic lineage: Adiponectin (ADPN), peroxisome proliferator‐activated receptor gamma (PPARγ) and CCAAT/enhancer‐binding protein α (CEBPα)

Primers used for qPCR			
Gene	Gene ID	Forward sequence	Reverse sequence
GAPDH	2597	ATC TTC CAG GAG CGA GAT	GGA GGC ATT GCT GAT GAT
SOX‐9	6662	GAG ACT TCT GAA CGA GAG	GGC TGG TAC TTG TAA TCC
ACAN	176	CAT CAC TGC AGC TGT CAC	AGC AGC ACT ACC TCC TTC
COL2	1280	AGC AGC AAG AGC AAG GAG AA	GTA GGA AGG TCA TCT GGA
COL1	1278	GTG GCA GTG ATG GAA GTG	CAC CAG TAA GGC CGT TTG
COL10α1	1300	GAA TGC CTG TGT CTG CTT	TCA TAA TGC TGT TGC CTG TTA
RUNX2	860	AGC AGC ACT CCA TAT CTC T	TTC CAT CAG CGT CAA CAC
OCN	632	GCA GAG TCC AGC AAA GGT G	CCA GCC ATT GATA CAG GTA GC
OPN	6696	ACG CCG ACC AAG GAA AAC TC	GTC CATA AAC CAC ACT ATC ACC TCG
OSX	121 340	CAG GCT ATG CTA ATG ATT ACC	GGC AGA CAG TCA GAA GAG
ALP	249	CCT TCA CTG CCA TCC TGT A	CGC CTG GTA GTT GTT GTG
ADPN	9370	CCG TGA TGG CAG AGA TGG	TAT ACA TAG GCA CCT TCT CCA G
PPARγ	5468	ACG AAG ACA TTC CAT TCA CAA GA	CTC CAC AGA CAC GAC ATT CAA
CEBPα	1050	CAA GAA CAG CAA CGA GTA	GTC ATT GTC ACT GGT CAG

### 2.9 Statistical analysis

For all data, a non‐parametric distribution was assumed, and the results are presented as mean ± standard deviation (SD). Cell viability after freezing was analyzed as single replicates by two‐way ANOVA followed by a Tukey's multiple comparison test. Alizarin red, Oil‐Red‐O, and GAG quantification were analyzed in technical duplicates by Kruskal‐Wallis tests and Dunn's multiple comparisons tests. Relative gene expression was analyzed as single replicates by two‐way ANOVA followed by a Tukey's multiple comparison test. Significance was set at *P* < .05 and all tests were performed using GraphPad Prism (version 7.0d for Mac OS X, GraphPad Software; San Diego, California). All graphs are indicated with N = number of biological replicates.

## 3 RESULTS

### 3.1 Cell Viability of cryo‐preserved hNPCs

**FIGURE 1:**
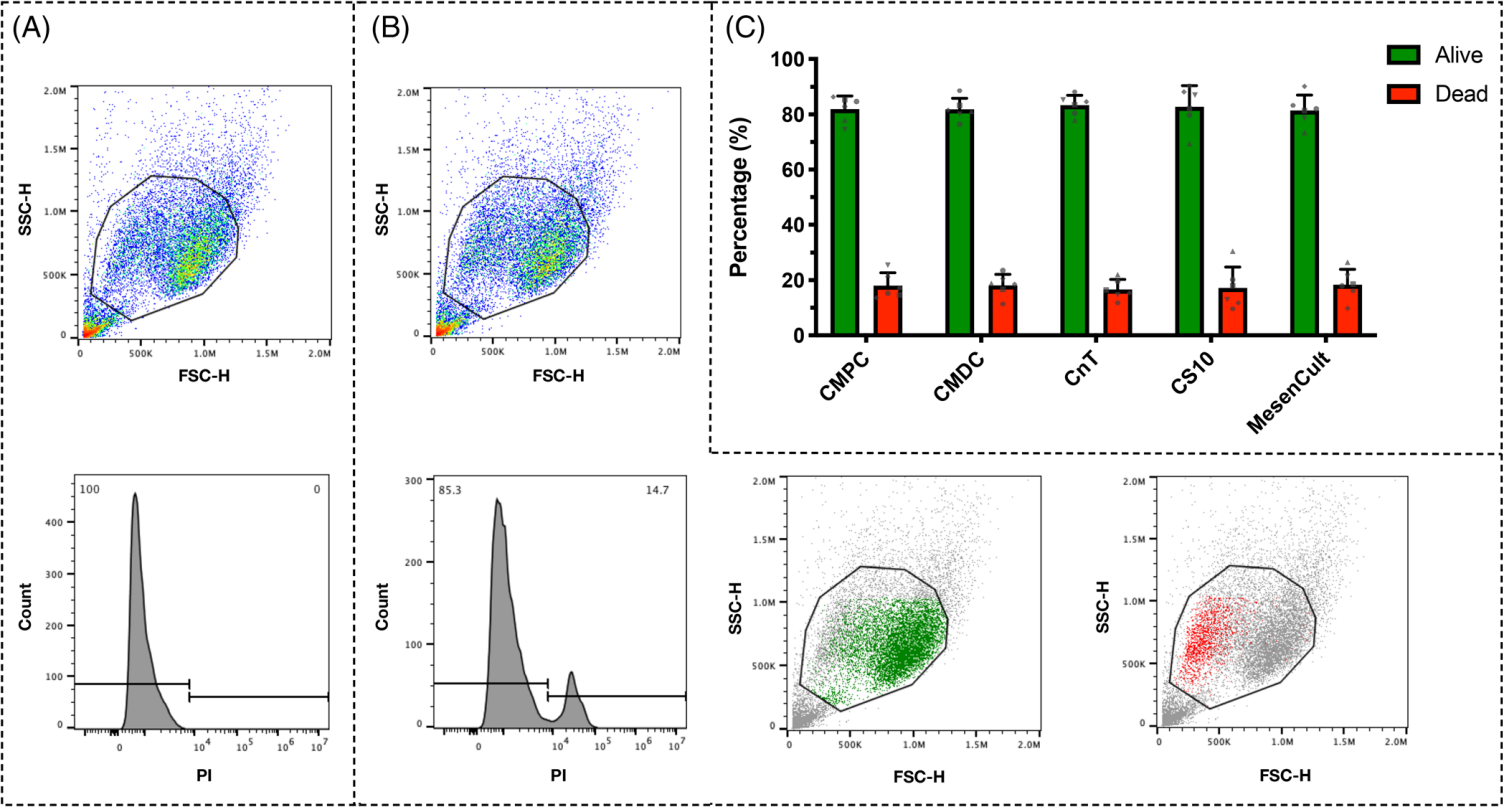
Flow cytometry analysis to assess the cell viability of hNPC populations after freezing them in five different cryopreservation media. A, Representative image of unstained hNPC population of one donor. B, Representative image of hNPC population of one donor stained with propidium iodide. Living cells are highlighted in green and dead cells in red. C, Graphical representation of living and dead NP cells that were previously frozen either in CMPC, CMDC, CnT, CS10, or MesenCult (mean ± SD, N = 6). The results show no significant differences when comparing the cell viability of the different groups with each other

To determine to most efficient cryopreservation medium for hNPCs in terms of viability and maintenance of the differentiation potential, hNPCs were resuspended either in the “commonly used” media (CMPC or CMDC) or in the commercially available media (CnT, CS10 or MesenCult), and then frozen. The hNPCs showed very similar cell viabilities independent of the cryopreservation media used and non‐significant differences when comparing the different groups with each other (CMPC: 82.0 ± 4.7%, CMDC: 81.9 ± 4%, CnT: 83.4 ± 3.6%, CS10: 82.8 ± 7.6% and MesenCult: 81.5 ± 5.5%). This indicates that “commonly used” cryopreservation media and commercially available media both performed the same in terms of cell viability (Figure 1).

### 3.2 Osteogenic differentiation

**FIGURE 2:**
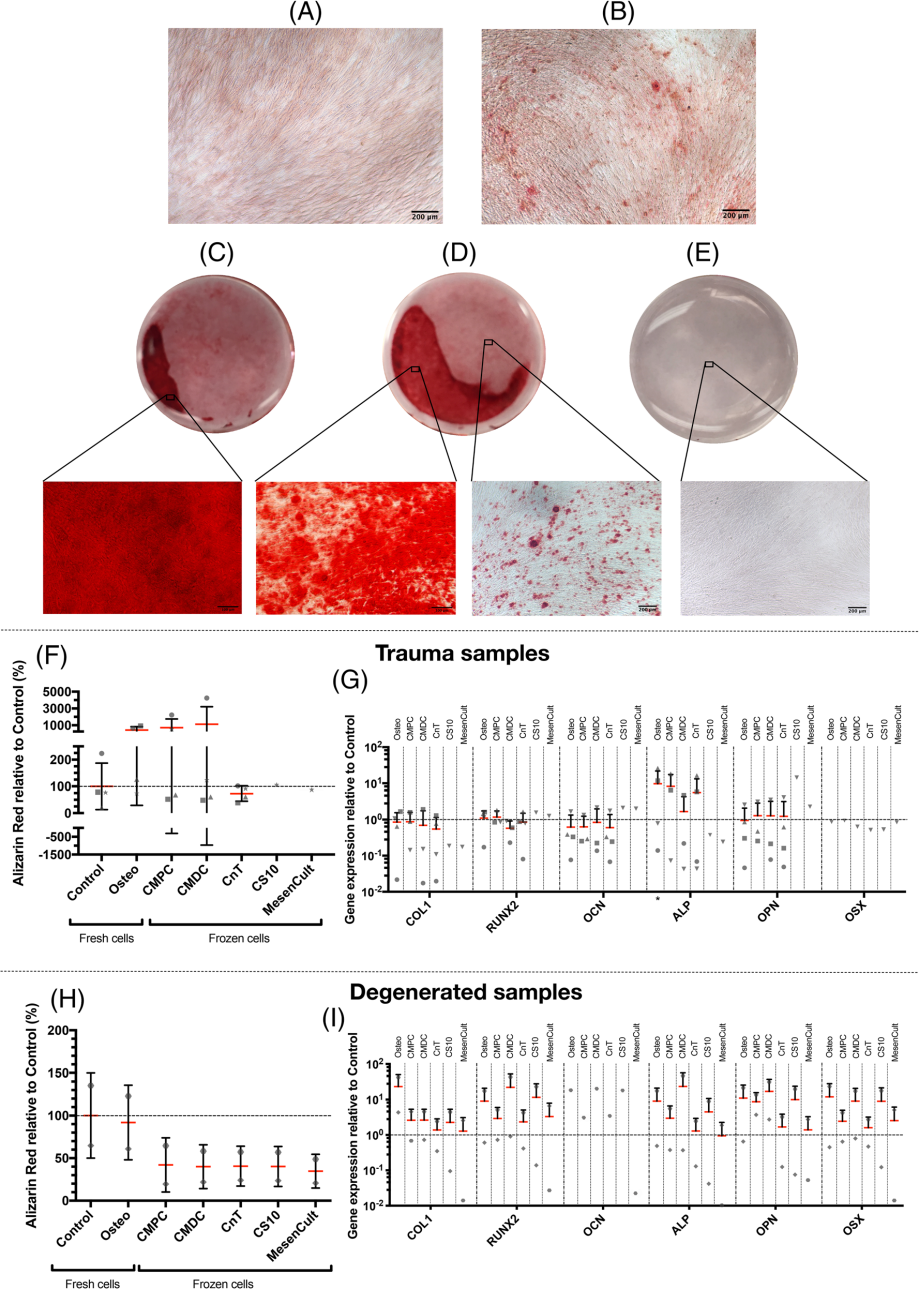
Representative images of the osteogenic differentiation assays. A, The hNPCs from trauma IVDs cultured in control medium or from the CS10 and MesenCult condition and then stained with Alizarin red. B, The hNPCs from trauma IVDs cultured in osteogenic inductive medium and stained with Alizarin red. C, Alizarin red stained single well of a 12‐well plate with hNPCs from trauma IVDs that were previously frozen in CMPC and then cultured in osteoinductive medium. D, Alizarin red stained single well of a 12‐well plate with hNPCs from trauma IVDs that were previously frozen in CMDC and then cultured in osteogenic inductive medium. E, Alizarin red stained single well of a 12‐well plate with hNPCs from trauma IVDs that were previously frozen in CnT and then cultured in osteogenic inductive medium. F, Relative quantification of the amount of solubilized Alizarin red from either fresh or previously frozen trauma hNPCs that were cultured in osteogenic inductive medium relative to cells cultured in control medium, which are set at baseline 100% (mean ± SD, N = 4 for Control, Osteo, CMPC, CMDC and CnT, N = 1 for CS10 and MesenCult). G, Osteogenic gene expression profile of trauma hNPCs relative to the control at baseline 1 (mean + SD, N = 4 for Control, Osteo, and CnT; mean + SD, N = 3 for CMPC and CMDC, N = 1 for CS10, MesenCult, and all osterix (OSX) results). H, Relative quantification of the amount of solubilized Alizarin red from either fresh or previously frozen hNPCs from degenerated IVDs that were cultured in osteogenic inductive medium relative to cells cultured in control medium, which are set at baseline 100% (mean ± SD, N = 2). I, Osteogenic gene expression profile of hNPCs from degenerated IVDs relative to the control at baseline 1 (mean + SD, N = 2 for all samples except OCN is N = 1 due to poor quality results of one donor). Nomenclature: Control: Fresh hNPCS cultured in control medium; Osteo: Fresh hNPCS cultured in osteogenic inductive medium; CMPC: hNPCs cryopreserved in CMPC and subsequently cultured in osteogenic inductive medium; CMDC: hNPCs cryopreserved in CMDC and subsequently cultured in osteogenic inductive medium; CnT: hNPCs cryopreserved in CnT and subsequently cultured in osteogenic inductive medium; CS10: hNPCs cryopreserved in CS10 and subsequently cultured in osteogenic inductive medium; MesenCult: hNPCs cryopreserved in MesenCult and subsequently cultured in osteogenic inductive medium. The following genes were analyzed: Collagen type I (COL1), runt‐related transcription factor 2 (RUNX2), osteocalcin (OCN), alkaline phosphatase (ALP), osteopontin (OPN) and osterix (OSX). *P < .05, no indication: *P* ≥ .05

From a histological point of view, differences could be observed between trauma cells cultured in the control medium and respective cells cultured in the osteogenic inductive medium. Sporadically, areas of mineralized matrix stained with Alizarin red could be seen on the osteogenic inductive samples, while no staining was visible in control samples (Figure 2A,B). Regarding the histological findings from previously frozen cells, samples frozen in CMPC and CMDC showed a considerable amount of Alizarin red stained areas when culturing them in osteogenic inductive medium. However, no sign of osteogenic differentiation could be found in the samples that were frozen in CnT, CS10, or MesenCult (Figure 2A‐E).

When analyzing the absorbance of solubilized Alizarin red stained matrix, fresh trauma samples cultured in osteogenic inductive medium and the ones frozen in CMPC and CMDC showed a relatively, but non‐significantly, higher amount of Alizarin red when comparing them to the control set at 100 ± 86.9% (Osteo: 435 ± 407%, CMPC: 718 ± 1029%, CMDC: 1120 ± 2085%, CnT: 73.3 ± 28.8%, CS10: 106%, MesenCult: 87.6%) (Figure 2F). Looking at the relative osteogenic gene expression profile of fresh trauma samples, only ALP showed a significant higher gene expression (Osteo: 9.7 ± 12.2‐fold increase). All other genes did not differ from the control in any significant way (Figure 2G).

On the other hand, hNPCs from degenerated IVDs did not show any microscopical evidence of matrix mineralization nor any indication of osteogenesis when the absorbance of solubilized Alizarin red was measured (Figure 2H). Furthermore, no osteogenic related gene was significantly up‐ or downregulated. Nevertheless, osteogenic related genes of degenerated samples were mostly stronger expressed, however not significantly, than trauma samples. This includes the genes COL1, RUNX2, OCN, OPN, and OSX (Figure 2I).

### 3.3 Adipogenic differentiation

**FIGURE 3:**
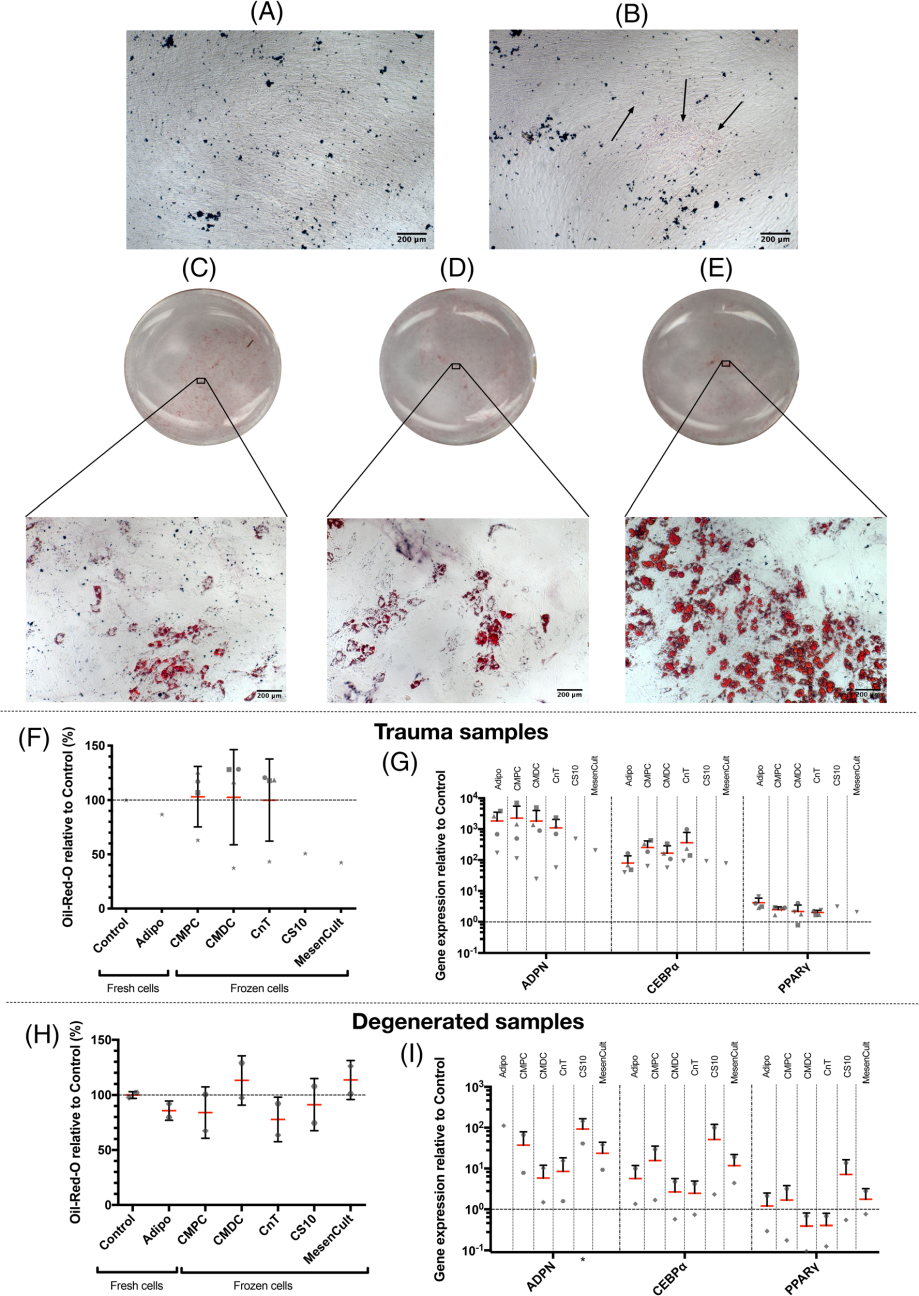
Representative images of the adipogenic differentiation assays. A, The hNPCs from trauma IVDs cultured in control medium or from the CS10 and MesenCult condition. B, The hNPCs from trauma IVDs cultured in adipogenic inductive medium. Lipid droplets are indicated with arrows. C, Oil‐Red‐O stained single well of a 12‐well plate with hNPCs from trauma IVDs that were previously frozen in CMPC and then cultured in adipogenic inductive medium. D, Oil‐Red‐O stained single well of a 12‐well plate with hNPCs from trauma IVDs that were previously frozen in CMDC and then cultured in adipogenic inductive medium. E, Oil‐Red‐O stained single well of a 12‐well plate with hNPCs from trauma IVDs that were previously frozen in CnT and then cultured in adipogenic inductive medium. F, Relative quantification of the amount of solubilized Oil‐Red from either fresh or previously frozen trauma hNPCs that were cultured in adipogenic inductive medium relative to cells cultured in control medium, which are set at baseline 100% (mean ± SD, N = 4 for CMPC, CMDC, and CnT, N = 1 for Control, Adipo, CS10, and MesenCult). G, Adipogenic gene expression profile of trauma hNPCs relative to the control at baseline 1 (mean + SD, N = 4 for Control, Adipo, CMPC, CMDC and CnT, N = 1 for CS10, and MesenCult). H, Relative quantification of the amount of solubilized Oil‐Red from either fresh or previously frozen hNPCs from degenerated IVDs that were cultured in adipogenic inductive medium relative to cells cultured in control medium, which are set at baseline 100% (mean ± SD, N = 2). I, Adipogenic gene expression profile of hNCPs from degenerated IVDs relative to the control at baseline 1 (mean + SD, N = 2). Nomenclature: Control: Fresh hNPCS cultured in control medium; Osteo: Fresh hNPCS cultured in adipogenic inductive medium; CMPC: hNPCs cryopreserved in CMPC and subsequently cultured in adipogenic inductive medium; CMDC: hNPCs cryopreserved in CMDC and subsequently cultured in adipogenic inductive medium; CnT: hNPCs cryopreserved in CnT and subsequently cultured in adipogenic inductive medium; CS10: hNPCs cryopreserved in CS10 and subsequently cultured in adipogenic inductive medium; MesenCult: hNPCs cryopreserved in MesenCult and subsequently cultured in adipogenic inductive medium. The following genes were analyzed: Adiponectin (ADPN), CCAAT/enhancer‐binding protein alpha (CEBPα) and peroxisome proliferator‐activated receptor‐gamma (PPARγ). *P < .05, no indication: *P* ≥ .05

Regarding the adipogenic differentiation potential of trauma hNPCs, lipid droplets were present with fresh cells, cultured with adipogenic inductive medium, as well as with cryopreserved (CMPC, CMDC, and CnT) cells. Numerous lipid droplets were visible even without the proper stain solution (Figure 3B‐E). In contrast, no lipid droplets were visible in the samples cultured with control medium or with trauma cells that were either frozen in CS10 or MesenCult (Figure 3A).

Due to challenging difficulties in staining a series of fresh cells using an incorrect Oil‐Red‐O staining solution, all adipogenic trauma samples were compared with a single control where the staining succeeded. Although a clear difference in adipogenic differentiation was visible between the control and some adipogenic inductive trauma samples (namely Adipo, CMPC, CMDC, and CnT), this could not be confirmed when measuring the absorbance of solubilized Oil‐Red‐O and as a result, no significant differences were found (Figure 3F).

In addition, no significant differences were seen for any condition with respect to the relative adipogenic gene expression profile of trauma hNPCs. Nevertheless, especially ADPN (Adipo: 1831.9 ± 1709.6‐fold increase, CMPC: 2280.9 ± 3241.2‐fold increase, CMDC: 1821.4 ± 21 821.6‐fold increase, CnT: 1096.0 ± 975.1 increase, CS10: 486.9‐fold increase, MesenCult: 205.3‐fold increase), but also CEBPα (Adipo: 80.0 ± 56.7‐fold increase, CMPC: 254.0 ± 162.3‐fold increase, CMDC: 166.3 ± 121.4‐fold increase, CnT: 364.0 ± 420.8 increase, CS10: 91.3‐fold increase, MesenCult: 77.6‐fold increase) displayed a strong trend for upregulated gene expression. However, PPARγ showed hardly any differences compared to the control (Figure 3G).

The quantification of solubilized Oil‐Red‐O from degenerated IVDs, hNPCs cultured in adipogenic inductive medium also revealed no significant differences compared to the control (Figure 3H). However, in contrast to the trauma cells, no lipid droplets could be found microscopically.

Despite the missing lipid droplets, significant upregulations of ADPN were observed for the CS10 condition (92.5 ± 73.1‐fold increase) with hNPCs derived from degenerated IVDs. Moreover, ADPN was consistently upregulated, although not significantly and also much weaker than the trauma samples (Adipo: 112.8‐fold increase, CMPC: 37.3 ± 41.7‐fold increase, CMDC: 5.9 ± 6.2‐fold increase, CnT: 8.5 ± 9.8‐fold increase, MesenCult: 23.6 ± 20.2‐fold increase). Furthermore, both CEBPα as well as PPARγ did not display any significant changes, even though CEBPα tended to be upregulated for all conditions. Thus, based on the qPCR results, it can be stated that cryopreservation had no significant influence on the cell's adipogenic differentiation potential, as no gene was significantly higher or lower expressed when comparing fresh cells with cryopreserved ones (Figure 3I).

### 3.4 Chondrogenic differentiation

**FIGURE 4:**
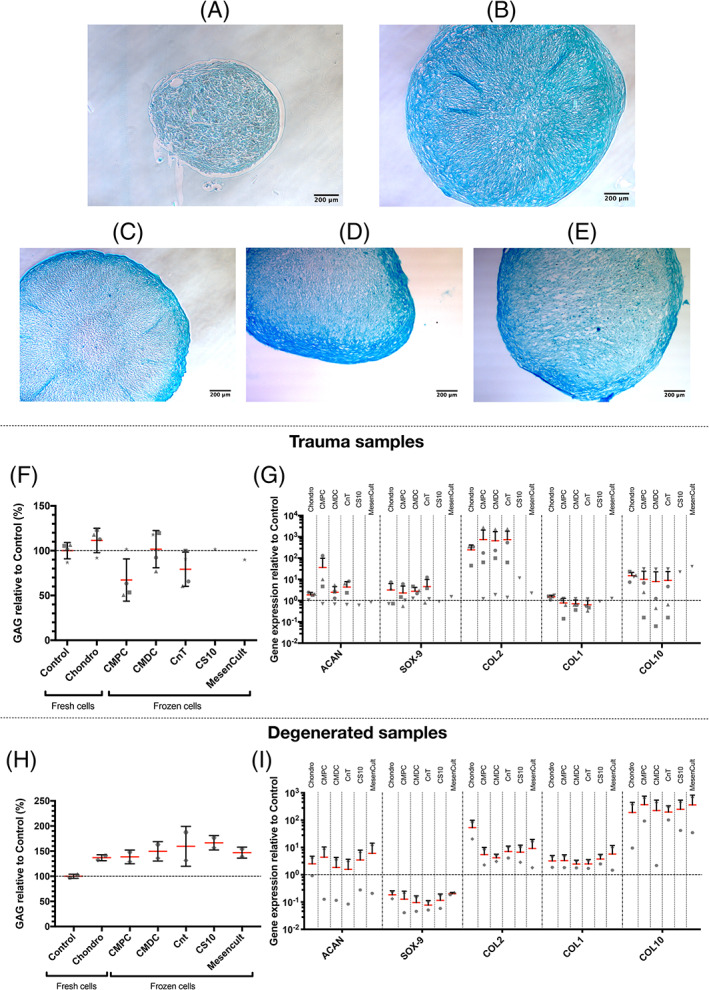
Representative images of the chondrogenic differentiation assays. A, The hNPCs from trauma IVDs cultured in control medium or from the CS10 and MesenCult condition and stained with Alcian blue. B, The hNPCs from trauma IVDs cultured in chondrogenic inductive medium and stained with Alcian blue. C, Alcian blue stained hNPCs from trauma IVDs that were previously frozen in CMPC and then cultured in chondrogenic inductive medium. D, Alcian blue stained hNPCs that were previously frozen in CMDC and then cultured in chondrogenic inductive medium. E, Alcian blue stained hNPCs from trauma IVDs that were previously frozen in CnT, CS10 or MesenCult and then cultured in chondrogenic inductive medium. F, Relative quantification of the amount of glycosaminoglycan released into the supernatant from either fresh or previously frozen trauma hNPCs that were cultured in chondrogenic inductive medium relative to cells cultured in control medium, which are set at baseline 100% (mean ± SD, N = 4 for Control, Chondro, CMPC, CMDC and CnT, N = 1 for CS10 and MesenCult). G, Chondrogenic gene expression profile of trauma hNPCs relative to the control at baseline 1 (mean + SD, N = 4 for Control, Chondro, CMPC, CMDC and CnT, N = 3 for COL2 with Chondro due to poor quality results of one donor, N = 1 for CS10 and MesenCult). H, Relative quantification of the amount of glycosaminoglycan released into the supernatant from either fresh or previously frozen hNPCs from degenerated IVDs that were cultured in chondrogenic inductive medium relative to cells cultured in control medium, which are set at baseline 100% (mean ± SD, N = 2). I, Chondrogenic gene expression profile of hNPCs from degenerated IVDs relative to the control at baseline 1 (mean + SD, N = 2). Nomenclature: Control: Fresh hNPCS cultured in control medium; Osteo: Fresh hNPCS cultured in chondrogenic inductive medium; CMPC: hNPCs cryopreserved in CMPC and subsequently cultured in chondrogenic inductive medium; CMDC: hNPCs cryopreserved in CMDC and subsequently cultured in chondrogenic inductive medium; CnT: hNPCs cryopreserved in CnT and subsequently cultured in chondrogenic inductive medium; CS10: hNPCs cryopreserved in CS10 and subsequently cultured in chondrogenic inductive medium; MesenCult: hNPCs cryopreserved in MesenCult and subsequently cultured in chondrogenic inductive medium. The following genes were analyzed: Aggrecan (ACAN), SOX‐9, collagen type II (COL2), collagen type I (COL1), and collagen type X (COL10). No indication: *P* ≥ .05

To analyze chondrogenic differentiation, 3D pellet samples were stained with Alcian blue. The chondrogenic pellets with cells from trauma patients were larger when examined by eye, and produced more GAG (stained in deep blue) than the control pellets (Figure 4A,B). Moreover, previously frozen cells, with subsequent culture in the chondrogenic inductive medium, showed results similar to fresh cells. Noteworthy, areas that were intensively stained with Alcian blue were predominantly located at the edges of the 3D pellet samples (Figure 4C‐E).

To study the chondrogenic differentiation of the 3D pellets, the concentration of GAG in the supernatant was determined after 21 days of culture. Even though the results are not significant, out of all conditions, fresh trauma cells that were cultured in chondrogenic inductive medium released the largest amount of GAG into the supernatant (Control: 100 ± 9.0%, Chondro: 111.4 ± 13.6%). Previously frozen chondrogenic trauma cells released similar or slightly less GAG than the control (CMPC: 67.3 ± 23.6%, CMDC: 101.7 ± 20.7%, CnT: 79.3 ± 19.2%, CS10: 101.7%, MesenCult: 90.1%) (Figure 4F). However, for the majority of conditions, a strong trend for COL2 expression was found compared to the control (Chondro: 244.1 ± 172.8‐fold increase, CMPC: 750.6 ± 1343.4, CMDC 654.7 ± 1093.7‐fold increase, CnT: 741.8 ± 1108.4‐fold increase, CS10: 11.3‐fold increase, MesenCult: 2.2‐fold increase). Contrarily, the other chondrogenic related genes ACAN and SOX‐9 did not differ from the control at all, and also COL1 showed no difference. Finally, COL10 was extensively expressed by trauma hNPCs, but also did not change significantly (Chondro: 14.8 ± 6.4‐fold increase, CMPC: 10.1 ± 14.1‐fold increase, CMDC: 7.9 ± 14.6‐fold increase, CnT: 9.0 ± 14.5 increase, CS10: 21.9‐fold increase, MesenCult: 39.5‐fold increase) (Figure 4G).

A further indication of how strong chondrogenic differentiation was can be seen when calculating the COL2 to COL1 ratio (Chondro: 162, CMPC: 966, CMDC: 926, CnT: 1170, CS10: 12.6, MesenCult: 1.8). Especially the Chondro, CMPC, CMDC, and CnT conditions show relatively high ratios and therefore indicate notable chondrogenic differentiation.

Regarding the chondrogenic differentiation potential of hNPCs from degenerated IVDs, all samples released a relatively higher amount of GAG into the supernatant. The results were not significant compared to the control set at 100 ± 4.0% (Chondro: 136.9 ± 6.0%, CMPC: 138.6 ± 13.7%, CMDC: 149.6 ± 19.3%, CnT: 159.6 ± 39.7%, CS10: 166.6 ± 14.2%, MesenCult: 147.0 ± 11.0%) (Figure 4H).

Concerning the expression of chondrogenic related genes with hNPCs from degenerated IVDs, again none of the genes were either significantly up‐ or downregulated. Only COL10 was noticeably higher expressed (Chondro: 191.2 ± 256.9‐fold increase, CMPC: 369.0 ± 389.9‐fold increase, CMDC: 226.1 ± 316.7‐fold increase, CnT: 198.7 ± 137.6 increase, CS10: 250.5 ± 295.1‐fold increase, MesenCult: 364.1 ± 465.3‐fold increase) (Figure 4I).

Also when calculating the COL2 to COL1 ratios, hNPCs derived from a degenerated IVD display much lower ratios than the trauma samples (Chondro: 16.6, CMPC: 1.7, CMDC: 1.7, CnT: 2.8, CS10: 1.8, MesenCult: 1.6).

## 4 DISCUSSION

### 4.1 Cryopreservation

This study showed that hNPCs can be cryo‐preserved with “commonly used” cryopreservation media just as well as with commercially available media without any significant differences concerning cell viability. Furthermore, no changes in cell proliferation were observed when comparing the different formulations. An explanation for the similar performance of all five cryopreservation media could be that all formulations are DMSO based and consequently have a very comparable mechanism of action. These results nicely show that researchers, working with hNPCs, can just as well cryo‐preserve their cells in “commonly used” cryo‐medium, which are potentially more affordable than commercially available media. Throughout the literature, many studies showed comparable results with different cell types regarding their viability after cryopreservation.^30^ To our knowledge, no study analyzed the effect of cryopreservation on the viability and the trilineage potential of hNPCs. However, Hiraishi et al. (2018) transplanted cryo‐preserved human NP cells into degenerated canine IVDs. They demonstrated, that thawed cells can arrest further degeneration by maintaining matrix features just as well as precultured cells.^33^

Nevertheless, DMSO‐based products still face some challenges for successful future cell‐based therapy. Despite the beneficial cryoprotective effects of DMSO, it is also known to be chemically toxic for cells, particularly in its liquid phase.^34^ In clinical use, DMSO has been shown to cause mild to severe adverse reactions with human tissues.^35^, ^36^, ^37^ Hence, it would be essential to properly remove any DMSO residues from frozen–thawed samples before considering regulatory affairs for cell therapy in patients in a good manufacturing practise (GMP)‐compliant environment.^38^

### 4.2 Osteogenic differentiation

With the osteogenic inductive medium, little to no evidence of osteogenesis was seen in fresh trauma cells as well as in samples previously frozen in one of the cryopreservation media. However, a high donor variability was seen in measurements of solubilized Alizarin red absorbance: Trauma hNPCs showed either strong mineralization of the ECM or no mineralization at all. Regarding the relative osteogenic gene expression profile, except for an up‐regulation of ALP, all other osteogenic related genes either did not significantly change or were even slightly downregulated when comparing them to their controls. Concerning the hNPCs derived from degenerated IVDs, no signs of ECM mineralization could be observed. However, osteogenic related genes were generally higher upregulated with degenerated samples than with trauma samples and therefore showing at least some evidence for osteogenic differentiation. Furthermore, the cryopreservation media tested have neither increased the hNPCs osteogenic potential nor weakened it.

Similar findings concerning the ossification potential of IVD cells of surgery tissue have also been reported by a recent report by Brown et al. (2018). They stated that hNPCs can calcify their ECM and can therefore become osteogenic in monolayer.^39^ Furthermore, Haschtmann et al. (2012) found in a rabbit disc organ culture modal that BMP2 and TGF𝛽‐3 could induce osteogenesis in differentiated AF cells.^40^ However, osteogenesis occurred inconsistently in our screened donors and only within a minority of the tested samples.^39^ These limited osteogenic capabilities are presumably either due to the existence of MSCs in the NP, so‐called NP‐MSCs described by Blanco et al. (2010), or this could be due to the presence of Tie2 positive progenitor cells in our samples. Previous studies proved that NP‐MSCs and Tie2 positive cells in human, mouse and bovine NP tissue can undergo osteogenic differentiation.^16^, ^19^ However, these results are based on the culture of very uniform Tie2 positive cell groups and not on a heterogeneous cell population, as used in our study. Furthermore, Tekari et al. (2016) showed that a mixed NP cell population manages to form some mineralization, but much less than pure Tie2 positive cells and therefore supports our findings.^19^

### 4.3 Adipogenic differentiation

Concerning the adipogenic differentiation potential of hNPCs, three out of four trauma samples, cultured in adipogenic inductive medium, were able to form lipid droplets, which could be microscopically detected. Even though the Oil‐Red‐O stain was unsuccessful in the majority of fresh hNPCs, the lipid droplets were still visible. Contrary to the microscopical findings, no significant differences could be observed when measuring the absorbance of the solubilized Oil‐Red‐O. This is probably due to the higher unspecific staining the control exhibited compared to the adipogenic inductive samples and consequently biased the results. When looking at the qPCR results from trauma cells, especially ADPN was highly expressed for all conditions and upregulated up to 2281 times compared to the control. However, as with the osteogenic samples, excessive donor variability was noted, which made any significant conclusions impossible. Also, CEBPα was notably expressed with values ranging between a 77‐fold‐ and a 364‐fold‐upregulation, depending on the condition. Only PPARγ did not show any evident changes compared to the control. Additionally, no significant changes between the gene expression of freshly isolated cells and cryopreserved cells were seen, indicating that cryopreservation did not have a negative influence on the adipogenic differentiation potential on trauma hNPCs, at least from a genetic point of view.

Regarding the adipogenic differentiation potential of hNPCs from degenerated IVDs, no sign of any lipid droplets could be microscopically observed. Even after measuring the Oil‐Red‐O absorbance of these samples, no significant differences between the conditions could be detected, neither with fresh cells nor with cryopreserved samples. However, the adipogenic related gene expression revealed a significant upregulation of ADPN for hNPCs that were previously frozen with CS10 and then cultured with inductive medium, which indicates at least a minimum of adipogenic differentiation. Furthermore, all genes were generally lower expressed with cells derived from degenerated discs compared to trauma ones. Moreover, as with the osteogenic samples, cryopreservation had no significant effect on the cell's differentiation potential.

These results seem to be very interesting, as on the one hand, we could partially confirm the findings of Blanco et al. (2010) who did not manage to produce any lipid droplets when culturing human NP cells from degenerated IVDs in adipogenic inductive medium.^16^ On the other hand, we successfully managed to produce lipid droplets with hNPCs derived from trauma IVDs, even after cryopreservation. Furthermore, the here presented results encourage the published data from Tekari et al. (2016), who showed adipogenic differentiation of bovine NPCs.^19^

### 4.4 Chondrogenic differentiation

Concerning the chondrogenic differentiation potential of trauma hNPCs, evidence for chondrogenic differentiation could be found with the Alcian blue staining for example. Especially at the border of the 3D pellets that were cultured in chondrogenic medium, a lot of GAG was produced and multiple chondrocyte‐like cells were visible. In contrast, the intensity of the Alcian blue stain in the control pellets was much weaker and the pellets were generally smaller than the chondrogenic pellets. Furthermore, relative gene expression of the trauma samples cultured in chondrogenic medium showed particularly a relatively high COL2 expression, which led to remarkable COL2 to COL1 ratios and therefore indicate notable chondrogenic differentiation. Nevertheless, large donor variability was present and consequently prevented any kind of significant differences between the control and the chondrogenic inductive samples. ACAN and SOX‐9 did not differ from the control and therefore, could not underline the chondrogenic potential of hNPCs as it was the case with COL2. Generally, the expression of COL1 did not change, neither in fresh cells nor in cryopreserved samples. However, against our hypothesis, also COL10 was higher expressed than the control, even though not significantly, indicating a hypertrophic state of the respective samples. This can be explained by the strong impact of TGF𝛽‐3, which we used in our chondrogenic inductive medium. Additionally, the upregulation of COL10 in the chondrogenic samples may also explain why the trauma samples cultured in chondrogenic inductive medium produced about the same amount of GAG on day 21 as the control. The peak of GAG production was probably already reached many days earlier.

Cells derived from degenerated IVDs showed a similar trend as the trauma samples in terms of gene expression. However, COL2 and SOX‐9 were generally lower expressed than the trauma cells. As seen with the trauma samples, COL10 was again non‐significantly upregulated and surprisingly, even stronger than with the respective trauma hNPCs. Additionally, ACAN and COL1 did not change or were expressed somewhat higher than the control, but also not significantly. Interestingly, all hNPCs that were cultured in chondrogenic inductive medium displayed higher but non‐significant accumulation of GAG in the supernatant, showing at least some evidence of chondrogenic differentiation from this point of view. Furthermore, it can be stated that release of GAG into the supernatant did not significantly change between fresh hNPCs and previously cryopreserved cells. This indicates that cryopreservation most likely did not influence the chondrogenic differentiation potential of these cells.

### 4.5 Study weaknesses and limitations

One limitation of this study is that only the cell viability and their differentiation potential before and after freezing was analyzed. Other parameters that could have been taken into consideration for the freezing assays would have been the cell proliferation rate, the cell's morphology, metabolism, or cell surface markers. However, a recent study involving BMSCs showed that cryopreservation does not affect the cell's surface markers, their proliferation rate, or their morphology.^31^ Even though BMSCs are not the same as hNPCs, similar results are likely to be expected with hNPCs. Another limitation concerning the freezing assay is that no long‐term data are available. Due to the strict and tight schedule of this study, cells could be frozen at −150°C only for a single week. Nevertheless, as the cellular metabolism almost comes to a standstill with so low temperatures and therefore, it can be assumed that no big changes are expected even after multiple weeks or even months of cryopreservation.^29^

An additional limitation of these types of donor studies was that trauma patients cannot be strictly be separated from patients with degenerated IVDs. We cannot fully exclude that trauma patients might have had an age appropriate degenerated IVD as for these patients no pre‐operative magnetic resonance image (MRI) was taken.

Another strong limitation of this study was that no Tie2 positive sorted human cells were investigated at any time point. Previous studies like Frauchiger et al. (2019) dealt with only Tie2 positive NP cells.^41^ However, human‐derived Tie2 positive cells were much fewer detected. This study also misses out to provide any evidence for CFUs in semi‐solid cellulose assay as it was not possible to perform these CFU assays in a meaningful way with human Tie2 positive cells. Bovine‐derived Tie2 positive cells could be much more isolated as these animals are very young and NP cells from several animals could be pooled. This enabled Frauchiger et al. (2019) and Sakai et al. (2018) to study the culture conditions of these cells much more in detail.^18^, ^41^

### 4.6 Future perspective

Future studies should focus on the responsible hNPC subpopulation responsible for their trilineage potential. Therefore, specific cell populations like Tie2 positive cells, CD73, CD90, and CD105 positive cells could be sorted and isolated from the remaining hNPCs and then separately tested for their trilineage potential. It is likely, that interaction and cooperation of progenitor cells, which equally contribute to the IVD's regeneration potential, is mainly responsible for the cell's stemness, rather than a single cell population. Finally, there is also the possibility that other undiscovered progenitor cells exist in the NP, which could have a huge impact on the stemness of hNPCs.

### 4.7 Conclusion

In this study, the freezing assay showed that “commonly used” cryopreservation media seem to be just as efficient as the commercially available media in terms of cell viability for hNPCs. Therefore, it is more economical using either a commonly applied cryo‐medium for progenitor cells or one for differentiated cells instead of a commercially available cryopreservation medium. Regarding the trilineage differentiation potential of hNPCs, trauma samples showed sporadic mineralization of their ECM but no indication of differentiation based on the results of osteogenic related genes. Concerning the trauma cell's adipogenic differentiation potential, the occasional production of lipid droplets was observed, however, when quantifying the solubilized Oil‐Red‐O, no significant differences were found compared to the control. Nevertheless, adipogenic related genes like ADPN and CEBPα showed relatively high, but non‐significant, upregulations. Trauma cell pellets cultured in chondrogenic inductive medium and subsequently stained with Alcian blue revealed a higher production of GAG than controls and COL2 to COL1 ratios of up to 1000. However, the amount of GAG released into the supernatant was similar for all samples. Regarding the samples derived from degenerated discs, the adipogenic and chondrogenic differentiation potential was generally less pronounced in hNPCs from degenerated IVDs than from trauma discs. Surprisingly, however, hNPCs from degenerated samples showed mostly a higher, but non‐significant, expression of osteogenic related genes than trauma cells, indicating a more successful differentiation towards osteogenesis than trauma samples.

Overall, cryopreservation did not broadly affect the differentiation potential of hNPCs, indicating that hNPCs are perfectly fine if they are being cryo‐preserved. However, extensive donor variations could be observed, which were maintained before and after freezing. Nevertheless, the results are promising and therefore clinical trials considering hNPCs for cell‐based IVD regeneration could be taken into consideration.

## CONFLICT OF INTEREST

The authors indicate no potential conflict of interest.

## AUTHORS' CONTRIBUTION

Andreas S. Croft: Acquisition of data, analysis and interpretation of data, drafting of the manuscript.

Julien Guerrero: Study conception and design, acquisition of data.

Katharina A. C. Oswald: Acquisition of data and reviewing of the manuscript.

Sonja Häckel: Acquisition of intervertebral discs, editing and reviewing of the manuscript.

Christoph E. Albers: Acquisition of intervertebral discs, editing and reviewing of the manuscript.

Benjamin Gantenbein: Study conception and design, analysis and interpretation of data, drafting and editing of the manuscript, and contributed the funding.
